# Synergistic Effect of Oxygen Vacancies and Ni Species on Tuning Selectivity of Ni/ZrO_2_ Catalyst for Hydrogenation of Maleic Anhydride into Succinic Anhydride and γ-Butyrolacetone

**DOI:** 10.3390/nano9030406

**Published:** 2019-03-11

**Authors:** Lili Zhao, Jianghong Zhao, Tianjie Wu, Min Zhao, Wenjun Yan, Yin Zhang, Haitao Li, Yongzhao Wang, Tiancun Xiao, Yongxiang Zhao

**Affiliations:** 1 Engineering Research Center of Ministry of Education for Fine Chemicals, School of Chemistry and Chemical Engineering, Shanxi University, Taiyuan 030006, China; lzhao@sxu.edu.cn (L.Z.); zhaojianghong@sxu.edu.cn (J.Z.); 201722907009@email.sxu.edu.cn (T.W.); sxuzhy@sxu.edu.cn (Y.Z.); htli@sxu.edu.cn (H.L.); catalyst@sxu.edu.cn (Y.W.); 2 Institute of Coal Chemistry, Chinese Academy of Sciences, Taiyuan 030001, China; zhaomin@sxicc.ac.cn (M.Z.); yanwenjun@sxicc.ac.cn (W.Y.); 3 Inorganic Chemistry Laboratory, Oxford University, Oxford, OX1 3QR, UK

**Keywords:** maleic anhydride, oxygen vacancies, selective hydrogenation, Ni/ZrO_2_

## Abstract

ZrO_2_ nanoparticles, ZrO_2_ (P) and ZrO_2_ (H), with different tetragonal phase contents, were prepared. ZrO_2_ (P) possessed higher tetragonal phase content than ZrO_2_ (H). Ni/ZrO_2_ catalysts (10% (*w*/*w*)), using ZrO_2_ (P) and ZrO_2_ (H) as supports, were prepared using an impregnation method, and were characterized using XRD, Raman, H_2_-TPR, XPS, and H_2_-TPD techniques. Their catalytic performance in maleic anhydride hydrogenation was tested. The Ni/ZrO_2_ (P) catalyst exhibited stronger metal-support interactions than the Ni/ZrO_2_ (H) catalyst because of its higher number of oxygen vacancies and the low-coordinated oxygen ions on its surface. Consequently, smaller Ni crystallites and a higher C=C hydrogenation activity for maleic anhydride to succinic anhydride were obtained over a Ni/ZrO_2_ (P) catalyst. However, the C=O hydrogenation activity of Ni/ZrO_2_ (P) catalyst was much lower than that of the Ni/ZrO_2_ (H) catalyst. A 43.5% yield of γ-butyrolacetone was obtained over the Ni/ZrO_2_ (H) catalyst at 210 °C and 5 MPa of H_2_ pressure, while the yield of γ-butyrolactone was only 2.8% over the Ni/ZrO_2_ (P) catalyst under the same reaction conditions. In situ FT-IR characterization demonstrated that the high C=O hydrogenation activity for the Ni/ZrO_2_ (H) catalyst could be attributed to the surface synergy between active metallic nickel species and relatively electron-deficient oxygen vacancies.

## 1. Introduction

**Figure 1:**
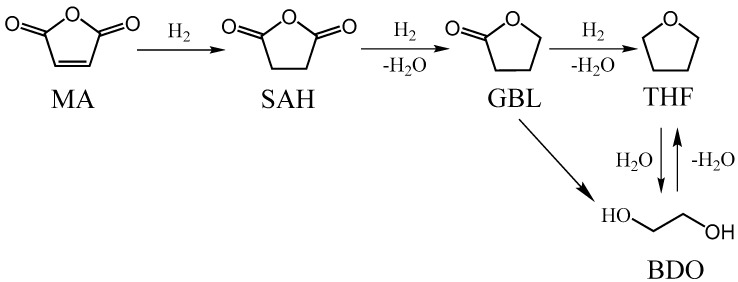
Reaction scheme of hydrogenation of maleic anhydride (MA).

Maleic anhydride (MA), as the third most important anhydride in commercial use, can be hydrogenated to produce succinic anhydride (SA), γ-butyrolacetone (GBL), 1,4-butanediol (BDO), and tetrahydrofuran (THF) products (Figure 1). SA is an important raw material of biodegradable plastic polybutylene succinate (PBS), and GBL is currently one of the most valuable and environmentally friendly media [1,2]. Hence, much attention has been focused on the selective hydrogenation of MA to SA or GBL. However, the process remains a challenge because of the coupled structure of the C=C and C=O bonds in MA molecules [3]. The coupled molecular structure leads to a delocalization of the electron density in the C=C and C=O bonds. This makes it difficult for the selectively hydrogenation of the C=C bond to obtain SA, or for the C=C and C=O bonds to obtain GBL. In addition, MA has a different molecular structure from other linear conjugated molecules, such as crotonaldehyde or acrolein, in that it is a compound with a five-membered cyclic structure. The special geometric structure of the reactant molecule will affect its adsorption mode on catalysts and the corresponding hydrogenation mechanism [4]. Therefore, the tailoring of high-activity and high-selectivity catalysts to obtain SA or GBL is an important but challenging subject.

In the current literature, the metal catalysts Pd, Ru, Cu, and Ni are widely accepted as being active in the hydrogenation of MA. Among them, the Ni catalyst has attracted a great deal of attention because of its high hydrogenation activity and low cost [5,6,7,8,9]. However, because of their weak hydrogenation ability towards the C=O bond of Ni [10,11], hydrogenation products are mostly mixtures of SA and GBL. Some valuable strategies have been employed to regulate the selectivity of Ni-based catalysts [12,13,14,15,16], such as introducing a second component as a promoter and modifying surface acid-base properties of supports. Results of experiments have also shown that these are effective regulation methods. In addition to these measures, the product distribution in MA hydrogenation could also be modulated by using reducible oxides as supports (e.g., CeO_2_ and TiO_2_) [17,18,19,20]. Our research group found that the Ni/CeO_2_ catalyst exhibited higher C=O hydrogenation activity when compared with the Ni/Al_2_O_3_ catalyst, and the superior catalytic activity in C=O hydrogenation was ascribed to the reduction of CeO_2_ [21]. In the hydrogenation of other carbonyl compounds and CO_2_ methanation reactions, it has been further found that the catalysts with abundant oxygen vacancies on the surface, such as CeO_2_, TiO_2_ and Co_3_O_4_ supported metal catalysts, exhibited superior catalytic performance in hydrodeoxygenation. The researchers concluded that the superior performances of these catalysts stemmed from the promotion of oxygen vacancies [22,23,24,25,26].

Recently, ZrO_2_ has received considerable attention and has been applied in a variety of reactions because of its amphoteric properties and the multiformity of its crystalline phases [27,28]. The morphology of ZrO_2_ was found to play a vital role in various catalytic reactions. Samson et al. found that when ZrO_2_ was present in the tetragonal phase, it showed a higher activity towards methanol synthesis from CO_2_ [29], whereas Rhodes et al. found that monoclinic ZrO_2_ was more active for methanol synthesis [30]. Amorphous ZrO_2_ was found to be beneficial for the dry reforming of the methane reaction [31]. The aforementioned experimental results, concerning the effects of the ZrO_2_ crystalline phase, seem to be contradictory. Whether the crystalline phase of ZrO_2_ is directly related to the catalytic activity of ZrO_2_ supported metal catalysts is unknown. Inspired by the reducible oxide-supported metal catalytic system, we speculate that the reason for the different catalytic behaviors of ZrO_2_-based catalysts might be the different surface structures of catalysts. Compared to CeO_2_ and TiO_2_, ZrO_2_ is more difficult to reduce. Whether oxygen vacancies are formed on the Ni/ZrO_2_ catalyst, and whether those oxygen vacancies affect catalytic performance in MA hydrogenation, until now, remains unclear. 

Therefore, in an effort to better understand the formation of oxygen vacancies on Ni/ZrO_2_ catalysts and the effect of oxygen vacancies on MA hydrogenation, we prepared two Ni/ZrO_2_ catalysts with different surface structures and tested their catalytic performance in MA hydrogenation. The results are presented in this paper.

## 2. Experimental Section

### 2.1. ZrO_2_ Preparation

ZrO_2_ (P) was prepared as follows [32]: firstly, a white precipitate was obtained by refluxing a mixture of a 0.5 M solution of zirconyl nitrate (ZrO(NO_3_)_2_ ·2H_2_O; >45.0% ZrO_2_, Beijing Chemicals, Beijing, China) at 100 °C for 240 h; during the process the solution pH value maintained at 1.5 by dropwise addition of ammonia solution. Then, the obtained precipitate was transferred into a Teflon-lined, stainless-steel autoclave (100 mL) and heated in an oven at 110 °C for 4 h. The final precipitate was washed with absolute alcohol until pH = 7, and then dried at 110 °C for 12 h before being calcined at 400 °C for 4 h.

ZrO_2_ (H) was prepared using a hydrothermal method [33]. It was synthesized at 140 °C under autogenous pressure for 2.5 h in a Teflon-lined stainless-steel autoclave (100 mL), which contained solutions (80 mL) of urea (CO(NH_2_)_2_; >99.9%, Beijing Chemicals, Beijing, China) and zirconyl nitrate (ZrO(NO_3_)_2_·2H_2_O; >45.0%, Beijing Chemicals, Beijing, China). The concentration of Zr^4+^ in the solutions was 0.2 M, and the urea/Zr^4+^ molar ratio was 10. The resulting precipitates were washed with absolute alcohol until they reached pH = 7, dried at 110 °C for 12 h, and then calcined at 400 °C for 4 h.

### 2.2. Ni/ZrO_2_ Preparation

NiO/ZrO_2_ catalysts were prepared using the impregnation method. Typically, 1.0 g of ZrO_2_ was added to an aqueous solution, consisting of 0.5476 g of nickel nitrate and 2.2 mL H_2_O, under vigorous stirring, after which the sample was dried at 120 °C for 12 h, and then calcined in air at 450 °C for 3 h. After the calcination treatment, the sample was denoted as NiO/ZrO_2_. Following that, the samples were reduced at 400 °C for 3 h in an H_2_ flow (30 mL/min), denoted as Ni/ZrO_2_.

### 2.3. Structure Characterizations

The nickel content in the Ni/ZrO_2_ catalysts was determined using inductively coupled plasma (ICP) on an iCAP 7400 ICP-OES (Thermo Fisher Scientific, Waltham, MA, USA).

The specific surface areas of the ZrO_2_ and ZrO_2_ supported catalysts were measured by N_2_ physisorption, at −196 °C, and using an ASAP-2020 instrument (Micromeritics, Atlanta, GA, USA).

X-ray diffraction (XRD) of the samples (ZrO_2_ support, NiO/ZrO_2_ and Ni/ZrO_2_) was performed using an X-ray diffractometer (Bruker D8 Advance, Karlsruhe, Germany) with Cu Kα radiation (λ = 1.54056 Å). The operating voltage and current were 40 kV and 40 mA, respectively.

Raman spectra were obtained using a Lab RAM HR Evolution Raman microscope (Horiba Scientific, Paris, France). The visible and UV Raman spectra were obtained using Ar^+^ (532 nm) and He-Cd lasers (325 nm) as excitation sources, respectively. A quantitative determination of the tetragonal phase x(T) content, present in each sample, was estimated using the following equation [34]:

X(T) = I(T)/[I(T) + I(M)]

where I(T) represents the added intensities of the two bands at ~148 and 269 cm^−1^, which are characteristic of the tetragonal phase, and I(M) denotes the added intensities of the two bands at 178 and 191 cm^−1^, and which are associated with the monoclinic phase.

The high-resolution transmission electron microscopy (HRTEM) images were measured on a JEOL JEM-2010 (Tokyo, Japan), which operated at 200 kV. Before taking the measurements, the NiO/ZrO_2_ samples were reduced at 400 °C for 3 h in H_2_ flow (30 mL/min) and then cooled to room temperature. Following that, the samples were transferred to a beaker containing anhydrous ethanol under N_2_ protection. Then, the samples were ultrasonically dispersed in ethanol and supported on a carbon-coated copper grid. High-angle annular dark-field scanning transmission electron microscopy (HAADF-STEM) and energy dispersive spectrometer (EDS) mapping images of the samples were obtained using a JEOL JEM-2010F (Tokyo, Japan) at 200 kV.

X-ray photoelectron spectroscopy (XPS) measurements were taken using a Kratos AXIS Ultra DLD spectrometer (Manchester, UK) with a monochromatic Al Kα (1486.6 eV) irradiation source. The X-ray gun operated at 150 W. The survey spectra were recorded with a pass energy of 160 eV, and the high-resolution spectra were recorded with a pass energy of 40 eV. The sampling area was 300 × 700 μm^2^. The binding energy was corrected by setting the C1s peak to 284.6 eV. For the ZrO_2_ samples’ test, they were placed into an XPS sample cell, which was then pumped down to 10^−8^ Pa before the spectra were recorded. For the test of the Ni/ZrO_2_ sample, the NiO/ZrO_2_ samples were first placed into an XPS sample cell, reduced at 400 °C for 3 h, and then cooled down to room temperature in H_2_ flow (30 mL/min). Subsequently, the sample cell was pumped down to 10^−8^ Pa, and then the spectra were recorded.

H_2_ temperature-programmed reduction (H_2_-TPR) was performed on a Micromeritics Auto Chem II 2920 (Atlanta, GA, USA) equipped with a thermal conductivity detector to determine the reducibility of the catalysts. First, 30 mg of Ni/ZrO_2_ sample were treated in Ar at 300 °C for 1 h and then cooled to 50 °C. Subsequently, the H_2_-TPR profiles were recorded while heating the samples in H_2_/Ar (10% *v*/*v*) with 50 mL/min of gas flow, from 50–700 °C at a ramp of 10 °C/min.

H_2_ temperature-programmed desorption (H_2_-TPD) measurements were carried out on the same apparatus as was used for the H_2_-TPR (Micromeritics Auto Chem II 2920, Atlanta, GA, USA). First, a 100 mg NiO/ZrO_2_ sample was first in situ reduced at 400 °C for 3 h in pure H_2_ and then cooled down to 50 °C. It was then purged with Ar for 1 h at 50 °C to remove the excess hydrogen adsorbed on the surface. H_2_/Ar (10% *v*/*v*) was then injected at 50 mL/min until saturation. Ar was used to flush the sample until the baseline was stable. H_2_-TPD profiles were recorded up to 700 °C at a heating rate of 10 °C/min.

In-situ FT-IR spectra of cyclohexanone were collected on a spectrometer (Bruker Tensor 27, Karlsruhe, Germany). 0.02 g NiO/ZrO_2_ sample were placed into an IR cell. Prior to the adsorption of cyclohexanone, the sample was reduced at 400 °C for 3 h in H_2_ flow (30mL/min) and then cooled to 210 °C. Following that, the IR cell containing the samples was pumped down to <6 ×10^−3^ Pa and a spectrum was recorded as the background. Gas cyclohexanone molecules were then introduced to the IR cell for the adsorption for 60 min. It was then desorbed, via vacuum pumping down to <6 ×10^−3^ Pa. The spectra were recorded with a resolution of 2 cm^−1^. 

### 2.4. Catalytic Activity Tests

The catalytic performances of the Ni/ZrO_2_ catalysts in the hydrogenation of MA were measured in a batch reactor (100 mL) with mechanical agitation at 210 °C and 5 MPa of H_2_ pressure. Before the test, the catalysts were pre-reduced using a stream of H_2_ (30 mL/min) in a quartz tube at 400 °C for 3 h and then cooled down to room temperature. Meanwhile, the MA (4.9 g) and THF (the purity of THF ≥ 99.99%, H_2_O ≤ 20 ppm) were charged in the autoclave. Then, the reduced catalyst (0.1 g) (40–60 mesh) was charged in the autoclave under N_2_ protection. Before each run, the autoclave was sealed and flushed with N_2_ three times and H_2_ five times to achieve a system pressure of 5 MPa. The reactor was heated to 210 °C, and the agitator operated at 400 rpm.

Different solvent such as 1,4-dioxane, cyclohexane were investigated. The results showed that the hydrogenation products were SA and GBL, with no THF and other products being detected. The carbon balance was between 95 and 105%. When THF was used as solvent, products SA and GBL are detected, and the carbon balance calculated based on the sum of SA and GBLwas between 95 and 105%. This suggest that there were no deep hydrogenation products like THF, BDO produced using THF as solvent for the present Ni/ZrO_2_ catalysts system.

The samples obtained from the reactor were analyzed using a gas chromatograph (Agilent, 7890B, Palo Alto, CA, USA) equipped with a DB-5 capillary column and FID detector. The conversion of MA and the selectivity to the product were calculated using the following equations:

*Conversion (MA) = (MA_in_ − MA_out_)/MA_in_ × 100%*

*Selectivity (i) = Product_i, out_/∑product_i, out_ × 100%*

where *MA_in_*, *MA_out_* and *Product_i,out_* represent the molar concentration of the inlet reactant, outlet reactant, and outlet product of species *i*, respectively.

## 3. Results

### 3.1. X-Ray Powder Diffraction (XRD) Patterns

**Figure 2:**
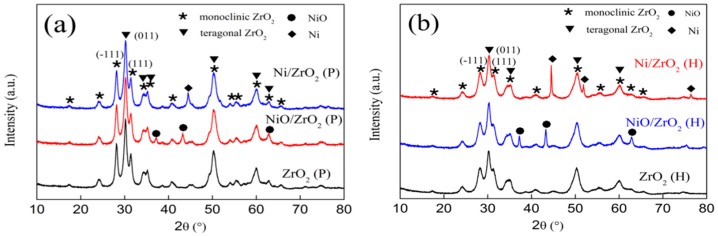
X-ray powder diffraction patterns of (**a**) ZrO_2_ (P) support and supported Ni catalysts and (**b**) ZrO_2_ (H) support and supported Ni catalysts.

The crystalline structures of the ZrO_2_ supports and the corresponding supported nickel catalysts were examined using XRD (Figure 2). This showed that the ZrO_2_ (P) and ZrO_2_ (H) possessed the characteristic lines of a mixture of monoclinic (JCPDS 65-1023) and tetragonal (JCPDS 81–1544) zirconia. The Rietveld method was used for diffraction peak deconvolution, and the calculated content of each phase is listed in Table 1 [35]. The results showed that there was a higher tetragonal phase (t-ZrO_2_) content for ZrO_2_ (P) than that for ZrO_2_ (H). The crystallite size of the ZrO_2_ (P) and the ZrO_2_ (H) calculated using the Scherrer equation are 16 and 10 nm, respectively. Based on “nanoparticle size effect” [36], the tetragonal phase can be stabilized at room temperature below a critical size (30 nm), which is due to the generation of excess oxygen vacancies; therefore, it could be supposed that more oxygen vacancies existed in ZrO_2_ (P) than in ZrO_2_ (H).

Figure 2 showed that new peaks appeared in the diffraction patterns of the NiO/ZrO_2_ (P) and NiO/ZrO_2_ (H) samples, which were attributed to crystalline NiO species (JCPDS 22-1189). The calculated mean crystallite size of NiO in the NiO/ZrO_2_ (P) was approximately 16 nm, which was smaller than the 26 nm of the NiO/ZrO_2_ (H) (Table 1). For the reduced Ni/ZrO_2_ (P) and Ni/ZrO_2_ (H) catalysts, the NiO characteristic diffraction peaks disappeared accompanied by the appearance of Ni characteristic peaks. The calculated mean crystallite size of Ni in the Ni/ZrO_2_ (P) was approximately 18 nm, while that of Ni/ZrO_2_ (H) grew to 40 nm. The crystallite size of ZrO_2_ was unchanged, even after calcination and reduction treatment for either the Ni/ZrO_2_ (P) or Ni/ZrO_2_ (H) catalysts.

### 3.2. Raman Spectra

**Figure 3:**
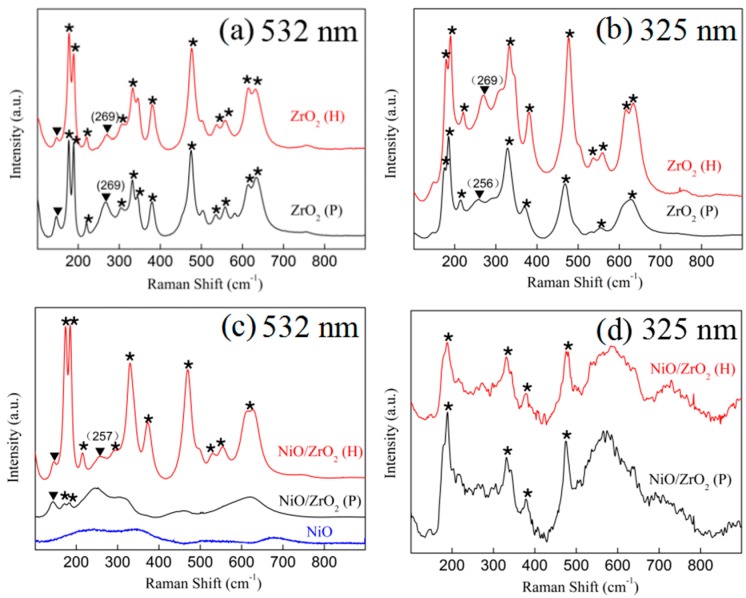
Raman spectra of ZrO_2_ supports and NiO/ZrO_2_ catalysts excited at 532 and 325 nm. ★ denotes monoclinic ZrO_2_; ▼ denotes tetragonal ZrO_2_. (**a**) Raman spectra of ZrO_2_ supports excited at 532 nm, (**b**) Raman spectra of ZrO_2_ supports excited at 325 nm, (**c**) Raman spectra of NiO/ZrO_2_ samples excited at 532 nm, (**d**) Raman spectra of NiO/ZrO_2_ samples excited at 325 nm.

Raman spectroscopy was used to further detect the microstructures of the samples due to its sensitivity to oxygen displacement and intermediate range order of the samples [37]. Excitation wavelengths of 325 and 532 nm were used to detect the phases, from the surface to the deeper inner part of the catalyst, which resulted from light absorption and light scattering {I∝(1/λ)^4^} [38]. When excited by the 532 nm laser (Figure 3a), both the ZrO_2_ (H) and ZrO_2_ (P) exhibited intense bands of Ag at 178 and 191 cm^−1^, Bg at 222, 333, and 382 cm^−1^, Ag at 476 cm^−1^, and Bg at 615 cm^−1^, which were assigned to the monoclinic ZrO_2_ (m-ZrO_2_) and the band Eg at 269 cm^−1^, which was ascribed to tetragonal ZrO_2_ (t-ZrO_2_) [39].

The quantitative determination of the tetragonal phase content of each sample is shown in Table 1. The content of tetragonal phase for ZrO_2_ (P) was 23%—higher than the 11% of ZrO_2_ (H)—showing that more oxygen vacancies existed in ZrO_2_ (P) than in ZrO_2_ (H). With 325 nm laser excitation (Figure 3b), both the ZrO_2_ (H) and the ZrO_2_ (P) revealed a monoclinic ZrO_2_ stretching peak. The difference is that the band of Eg was at 269 cm^−1^ for ZrO_2_ (H), while a band centered at 256 cm^−1^ appeared for ZrO_2_ (P). The peak, centered at 269 cm^−1^, was a typical characteristic peak for the tetragonal phase and showed the characteristics of an asymmetric Zr-O-Zr stretching mode [40]. It was reported that the shift of this peak to a lower wavenumber is due to the movement of oxygen [41]. The calculated results of the XRD and the 532 nm Raman spectroscopy showed that the tetragonal phase content in the ZrO_2_ (P) was higher than that in the ZrO_2_ (H). Thus, the peak at 256 cm^−1^ is ascribed to a decrease in the symmetry of the tetragonal phase structure, which was caused by the higher number of oxygen vacancies.

As shown in Figure 3d, the NiO/ZrO_2_ (H) and NiO/ZrO_2_ (P) catalysts exhibited typical monoclinic phase behavior with no discernible differences at 325 nm laser excitation. Upon being excited by a 532 nm laser, stronger and more well-defined Raman peaks were obtained (Figure 3c), which gave accurate overall structure information of the NiO/ZrO_2_ samples. For the NiO/ZrO_2_ (H), the Raman spectrum is dominated by strong bands which were attributed to m-ZrO_2_ and a less prominent broad band at 257 cm^−1^. The slight shift of the band from 269 cm^−1^ for ZrO_2_ (H) to 257 cm^−1^ for NiO/ZrO_2_ (H) could be ascribed to the increase in the number of oxygen vacancies. This was most likely caused by the interaction between nickel species and ZrO_2_ (H). The NiO/ZrO_2_ (P) spectrum showed only broad continuum lines with poorly defined bands maxima at 245, 448 and 620 cm^−1^. which were attributed to a breakdown of the wave-vector selection rule by translational disorder caused by the random substitution of vacancies or cations [42]. This meant that the interaction between the nickel species and ZrO_2_ (P) support was stronger, which led to a disordered tetragonal structure.

A comparison of the XRD and Raman results showed no discernible change in the ZrO_2_ structure after the nickel loading in the XRD, whereas the Raman spectra exhibited significant changes in the tetragonal structure for the NiO/ZrO_2_ (P) and NiO/ZrO_2_ (H) catalysts, which also provides strong evidence that Raman is more sensitive to the intermediate range structures, while XRD characterizes the long-range ordering of the structures.

### 3.3. High-Resolution Transmission Electron Microscopy (HRTEM) Images

**Figure 4:**
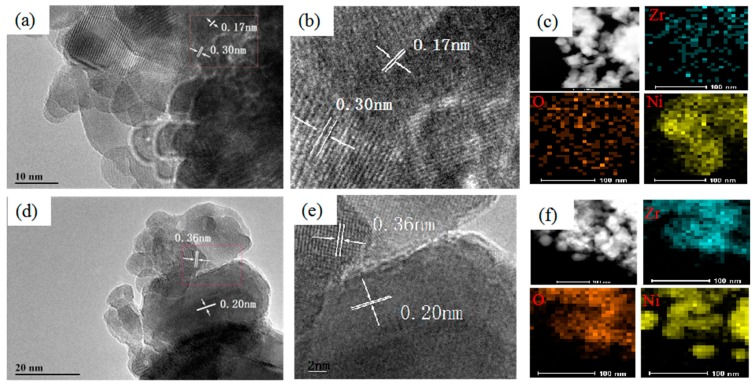
High-resolution transmission electron microscopy (HRTEM) images of (**a**) Ni/ZrO_2_ (P) and (**d**) Ni/ZrO_2_ (H). Enlarged selected area HRTEM images of (**b**) Ni/ZrO_2_ (P) and (**e**) Ni/ZrO_2_ (H). HAADF-STEM images and corresponding EDS elemental mapping images of (**c**) Ni/ZrO_2_ (P) and (**f**) Ni/ZrO_2_ (H).

HRTEM images of the Ni/ZrO_2_ catalysts are shown in Figure 4. Metallic Ni with lattice spacings of 0.17 and 0.20 nm were observed for both the Ni/ZrO_2_ (P) and Ni/ZrO_2_ (H) catalysts. It is noticeable that the interface between the Ni nanoparticles and the ZrO_2_ (P) substrate was a coalesced heterostructure, as shown in Figure 4a (and enlarged in Figure 4b). The Ni nanoparticles are embedded into a large ZrO_2_ (P) substrate, with an irregular borderline emerging in the disordered interface region. The lattice spacing of the t-ZrO_2_ (101) increased from 0.29 to 0.30 nm, indicating that some of the nickel ions incorporated into the t-ZrO_2_ lattice through Ni^2+^ dissolution into ZrO_2_ (P) or were located at the interstitial sites, resulting in the formation of a Ni-O-Zr structure, and thereby causing lattice expansion [43]. This shows that there was a strong interaction between the closely contacted nickel species and the t-ZrO_2_ for the Ni/ZrO_2_ (P), which agrees with the Raman results. However, unlike the Ni/ZrO_2_ (P), there were large Ni particles surrounded by small ZrO_2_ (H) particles for the Ni/ZrO_2_ (H) catalyst without the appearance of coalesced structures, as shown in Figure 4d, but with a loosely contacted region at the interfaces (Figure 4e). As shown in Figure 4c,f, smaller nickel particles were uniformly dispersed on the ZrO_2_ substrate for the Ni/ZrO_2_ (P) catalyst, while the nickel particles were aggregated around the ZrO_2_ particles for the Ni/ZrO_2_ (H) catalyst. 

### 3.4. H_2_ Temperature-Programmed Reduction (H_2_-TPR)

**Figure 5:**
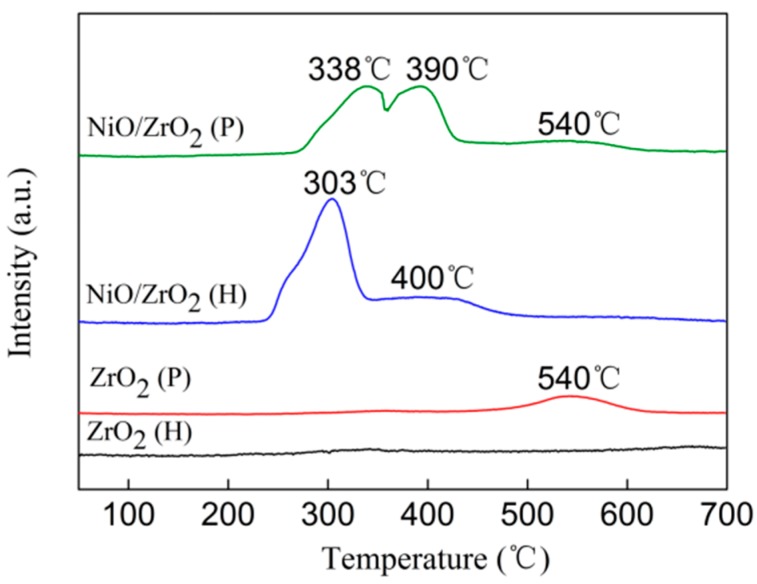
H_2_-TPR profiles of ZrO_2_ supports and NiO/ZrO_2_ catalysts.

The H_2_-TPR profiles of ZrO_2_ (P), ZrO_2_ (H), and their corresponding supported nickel catalysts are presented in Figure 5. No reduction peaks were observed for ZrO_2_ (H), while ZrO_2_ (P) exhibited an obvious reduction peak at 540 °C, indicating that ZrO_2_ (P) was more easily reduced. It was reported that surficial O atoms at low-coordinated sites are easily removed, and that oxygen vacancies facilitate the activation and transportation of active oxygen species, thereby promoting the reducibility of ZrO_2_ [44]. From this perspective, the reducibility of ZrO_2_ (P) originated from its special surface structure, more low-coordinated oxygen ions and oxygen vacancies on the surface of ZrO_2_ (P). Furthermore, these low-coordinated sites introduce defective states in the band gap and enhance the interaction with the deposited metal catalysts [44,45]. This is consistent with our Raman and HRTEM experiment results—i.e., nickel species have a stronger interaction with the ZrO_2_ (P) support. Furthermore, it can be understood that the stronger interaction between nickel species and ZrO_2_ (P) originates from the abundance of low-coordinated oxygen ions and oxygen vacancies on the surface of ZrO_2_ (P).

The NiO/ZrO_2_ (H) showed a sharp reduction peak centered at 303 °C with a minor shoulder peak at 400 °C. The H_2_ uptake peak at 303 °C was attributed to the reduction of large NiO that had weak interactions with the support [46]. For the NiO/ZrO_2_ (P) sample, three H_2_ uptake peaks were observed at 338, 390, and 540 °C. The first peak was assigned to the reduction of NiO particles with weak interactions with the support. The second peak was related to the NiO exhibiting a relatively strong interaction with the support. Compared to the profile of the ZrO_2_ (P), the reduction peak at 540 °C could be attributed to the reduction of ZrO_2_ [46]. The reduction temperature of the NiO/ZrO_2_ (P) was much higher than that of the NiO/ZrO_2_ (H), indicating the presence of strong interactions between the nickel species and the ZrO_2_ (P). The strong interaction between the nickel species and support could hinder the migration of nickel species during the calcination and reduction procedure. In this way, smaller Ni particles could be obtained in the NiO/ZrO_2_ (P) sample. These findings were in in line with the results of XRD and HRTEM.

### 3.5. XPS Characterization

**Figure 6:**
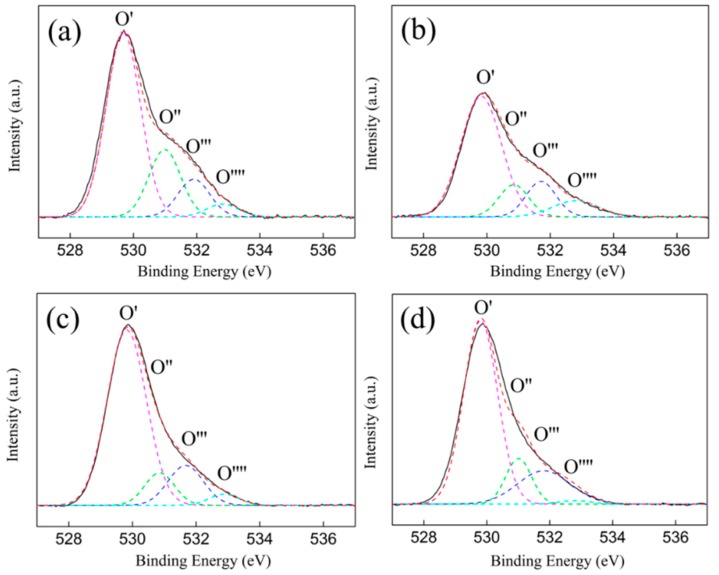
O 1s XPS spectra of the ZrO_2_ supports and Ni/ZrO_2_ catalysts: (**a**) ZrO_2_ (P), (**b**) ZrO_2_(H), (**c**) Ni/ZrO_2_ (P), and (**d**) Ni/ZrO_2_ (H).

To explore the properties of the oxygen vacancies on the ZrO_2_ support and their changes after loading nickel, XPS was conducted. It is widely accepted that a neutral O vacancy introduces two extra electrons in the lattice, which can be localized either in the created vacancy or in nearby cation sites. In ZrO_2_, the extra charge is trapped in the vacancy site rather than reducing the nearest Zr ions. Hence, there were three favored charge states for oxygen vacancies existed on surface of the ZrO_2_: a neutral oxygen vacancy with the two electrons remaining at the oxygen vacancy, a singly charged oxygen vacancy, and a doubly charged oxygen vacancy [44]. Due to the decrease in charge density, an increase in the O1s binding energy for the singly-charged oxygen and the doubly-charged oxygen vacancies is inevitable, while the neutral oxygen vacancy peak likely lies at or near the same position as the lattice oxygen ion peak. In K. T. Leung’s work on XPS fitting [47], oxygen vacancies were fitted into two types of oxygen vacancies, and the changes in the oxygen vacancies’ electronic properties were expounded more clearly. In line with this earlier research, the O1′s spectra from the samples in this study were analyzed, using curve fitting and four peaks were assigned to the lattice oxygen (O’), singly charged oxygen vacancies (O’’), doubly charged oxygen vacancies (O’’’), and the hydroxyl or/and carbonates groups (O’’’’) on ZrO_2_ (Figure 6).

Table 2 shows summary details for the binding energy and the surface atomic concentration that was calculated by integrating different oxygen species’ peak areas. Two forms of oxygen vacancies were centered at 531.0 (BE_2_) and 531.9 eV (BE_3_), which corresponded to the singly and doubly charged oxygen vacancies on the ZrO_2_ (P), respectively. For the ZrO_2_ (H), the binding energies of the singly and doubly charged oxygen vacancies were centered at 530.8 (BE_2_) and 531.7 eV (BE_3_), respectively. The different binding energies, shown for the same types of oxygen vacancies on the ZrO_2_ (P) and the ZrO_2_ (H), illustrate that the electron properties of the oxygen vacancies on the ZrO_2_ (P) and the ZrO_2_ (H) are different. The oxygen vacancy concentration of the ZrO_2_ (P) was 33.1%—much higher than that of the ZrO_2_ (H) (24.5%). This was consistent with the XRD and Raman results. A comprehensive analysis of the above characterization results shows that the ZrO_2_ support, with a higher concentration of electron-deficient oxygen vacancies and low coordination oxygen ion on the surface, demonstrates much stronger interactions with the Ni species.

After nickel loading, the binding energy of O’’ for the Ni/ZrO_2_ (P) shifted from 531.0 to 530.8 eV, and the binding energy of O’’’ shifted from 531.9 to 531.7 eV. Meanwhile the total oxygen vacancy concentration decreased significantly, from 33.1% to 24.7%. This was potentially because a small amount of the nickel species entered into the oxygen vacancies, and the oxygen vacancies bore extra charges for the charge balance in the Ni-O-Zr like structure [48]. However, the binding energy of O’’ and O’’’ increased from 530.8 and 531.7 eV to 531.0 and 531.9 eV, respectively, after ZrO_2_ (H) loading nickel. Additionally, the total oxygen vacancy concentration increased slightly when compared to that of the ZrO_2_ (H) support. This was because the Ni^0^ particles, which were first reduced, promoted the generation of additional oxygen vacancies at the Ni/ZrO_2_ interface and caused local structural deformation around the vacancy, which has been observed in Raman characterization [49]. Furthermore, the oxygen vacancies that were promoted by the Ni^0^ exhibited lower charge densities, and were different from the inherent oxygen vacancies of the ZrO_2_ (H).

**Figure 7:**
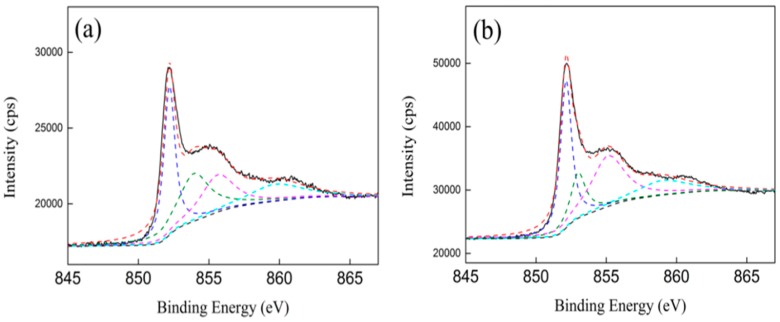
Ni 2p XPS spectra of Ni/ZrO_2_ catalysts: (**a**) Ni/ZrO_2_ (P) and (**b**) Ni/ZrO_2_ (H).

Figure 7 shows the Ni 2p XPS spectra of the Ni/ZrO_2_ catalyst. Three different chemical states of nickel were found in the Ni 2p_3/2_ XPS spectra. In the Ni 2p_3/2_ XPS spectra, the binding energy of the Ni^0^ was situated at 852.2 eV [50], and the relative amount of Ni^0^ species on the Ni/ZrO_2_ (P) was 57.8%, which was almost equal to that on the Ni/ZrO_2_ (H) (58.4%) (Table 3). Two other peaks appeared for the Ni/ZrO_2_ (P): one at 853.9 eV was attributed to Ni^2+^, in the form of NiO, and the other, at 855.6 eV, was attributed to Ni^2+^, in the form of oxide and hydroxide phases [50]. For the Ni/ZrO_2_ (H), the corresponding binding energy of the above two peaks shifted to 853.0 and 855.1 eV, respectively. The higher binding energy of the nickel species in the Ni/ZrO_2_ (P) further confirmed that nickel species have a stronger interaction with ZrO_2_ (P).

### 3.6. Catalytic Performances of Ni/ZrO_2_ (P) and Ni/ZrO_2_ (H) Catalysts

**Figure 8:**
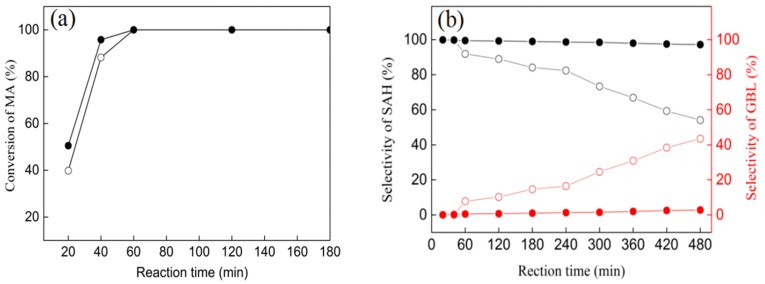
The MA conversion (**a**) and selectivity of SA and GBL (**b**) over the Ni/ZrO_2_ (P) (solid symbols) and Ni/ZrO_2_ (H) (open symbols) catalysts at 210 °C under 5 MPa for 480 min.

The hydrogenation of MA over the Ni/ZrO_2_ (P) and Ni/ZrO_2_ (H) catalysts was performed in a batch reactor at 210 °C and 5.0 MPa of H_2_ pressure (Figure 8). The hydrogenation products were SA and GBL, with no other deep hydrogenation products or by-products being detected. Figure 8a shows that the Ni/ZrO_2_ (P) exhibited a slightly higher MA conversion at the initial reaction stage. With a reaction time of 20 min, the conversion of MA for the Ni/ZrO_2_ (P) catalyst was 51%, while that for the Ni/ZrO_2_ (H) was 40%. MA conversion reached 100% for the Ni/ZrO_2_ (P) and Ni/ZrO_2_ (H) catalysts within 60 min. The product distribution was significantly different for the two catalysts, in the prolonged hydrogenation process which took 480 min. As shown in Figure 8b, the initial hydrogenation product was SA for the two catalysts before MA conversion reached 100%. As the time of the stream increased, the selectivity of the SA decreased gradually. This was accompanied by a gradual increase in the GBL selectivity over the Ni/ZrO_2_ (H) catalyst. The GBL selectivity reached up to as much as 43.5% after 480 min. However, the SA selectivity decreased slightly over Ni/ZrO_2_ (P) catalyst, and the GBL selectivity was only 2.8% after 480 min over Ni/ZrO_2_ (P) catalyst.

The above results showed that hydrogenation of MA to GBL was carried out in two successive reaction processes over the Ni/ZrO_2_ catalysts—the hydrogenation of MA to SA, followed by the hydrogenation of C=O in SA to produce GBL. Before the MA conversion reached 100%, the hydrogen product was SA for the Ni/ZrO_2_ (P) and Ni/ZrO_2_ (H) catalysts. Furthermore, the Ni/ZrO_2_ (P) catalyst exhibited a slightly higher C=C bond hydrogen activity. However, the C=O hydrogenation activity of the Ni/ZrO_2_ (P) was much lower than that of the Ni/ZrO_2_ (H) catalyst. A 43.5% yield of GBL was obtained over the Ni/ZrO_2_ (H) catalyst at 210 °C and 5 MPa of H_2_ pressure, while only 2.8% yield of GBL was obtained over the Ni/ZrO_2_ (P) catalyst under the same reaction conditions. Even enhancing the reaction temperature or reaction pressure, the Ni/ZrO_2_ (H) catalyst exhibited enhanced activity in C=O hydrogenation, whereas the Ni/ZrO_2_ (P) catalyst still showed extremely low C=O hydrogenation activity (Table 4).

### 3.7. H_2_ Temperature-Programmed Desorption (H_2_-TPD)

**Figure 9:**
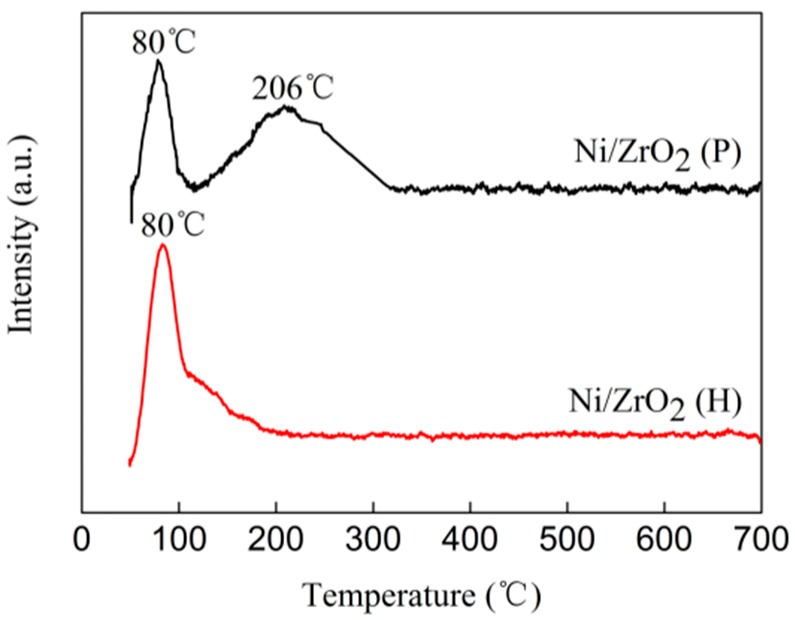
H_2_-TPD profiles of Ni/ZrO_2_ catalysts.

For the hydrogenation reaction catalyzed by metallic nickel, the surface area of nickel plays a crucial role, as hydrogen dissociation always occurs on active metallic Ni^0^ sites. To explore the reasons for the significant differences in C=O hydrogenation activity between the Ni/ZrO_2_ (P) and Ni/ZrO_2_ (H) catalysts, H_2_-TPD characterization was conducted to investigate the activating hydrogen ability of the two catalysts. As shown in Figure 9, only one peak centered at 80 °C was detected for the Ni/ZrO_2_ (H) catalyst. This was assigned to desorption of H which was weakly adsorbed on the Ni surface [51]. Compared to the Ni/ZrO_2_ (H) catalyst, the Ni/ZrO_2_ (P) exhibited two H desorption peaks: at lower temperature (~80 °C) and higher temperature (~206 °C) H desorption peaks. The new peak at the higher temperature originated from the more strongly chemisorbed H [51]. According to the literature [50], H_2_-TPD peaks at temperatures below 300 °C can be attributed to the desorption of H from the surface of Ni. Our calculations showed that the amount of H desorption for Ni/ZrO_2_ (P) was 1.12 times that of Ni/ZrO_2_ (H) below 210 °C, which is consistent with the assumption that smaller Ni particles provide more hydrogen activation sites.

Generally, the more adsorption and activation hydrogen sites on the catalyst, the higher of the hydrogenation activity. The H_2_-TPD characterization results showed that the Ni/ZrO_2_ (P) catalyst possessed more hydrogen adsorption and activation sites, yet it exhibited only slight C=O hydrogenation activity. Therefore, it is surmised that the major reason for the different C=O hydrogenation activities between the two catalysts was their different adsorption and activation abilities for C=O.

### 3.8. In-Situ FT-IR Spectra

**Figure 10:**
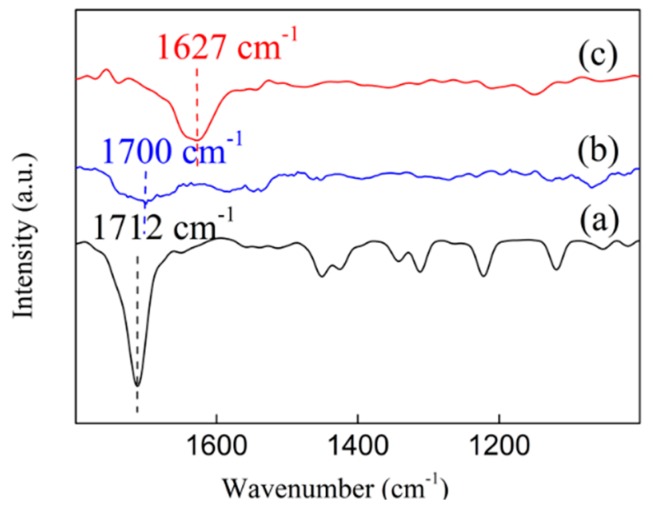
In-situ FT-IR of cyclohexanone (**a**) pure cyclohexanone, (**b**) cyclohexanone adsorbed on Ni/ZrO_2_ (P), (**c**) cyclohexanone adsorbed on Ni/ZrO_2_ (H).

In order to explore the adsorption and activation abilities towards the C=O of the catalysts, in-situ FT-IR was investigated over two Ni/ZrO_2_ catalysts using cyclohexanone as a probe molecule. In Figure 10, the peak centered at 1712 cm^−1^ was assigned to the C=O stretching vibration of pure cyclohexanone. Compared with the pure cyclohexanone, a significant redshift of the C=O stretching vibration peak occurred over the Ni/ZrO_2_ (H) catalyst even down to 1627 cm^−1^, while the peak corresponding to the C=O bond for the Ni/ZrO_2_ (P) catalyst was weak and located at 1700 cm^−1^. The two catalysts exhibited significantly different adsorption and activation properties towards C=O bonds. The much larger shift of the C=O bond indicated the weakening of the C=O bond and thereby the activation of them on surface of Ni/ZrO_2_ (H) catalysts [52]. No obvious shift occurred on the Ni/ZrO_2_ (P) catalyst, and the peak area was extremely small, indicating that the Ni/ZrO_2_ (P) catalyst had very weak adsorption and activation abilities towards C=O.

## 4. Discussion

Generally, the catalytic performance of supported catalysts is intrinsically linked to the active metal sites and supports, including the active metal and metal-support interactions. As concerns hydrogenation reaction, it is well documented that H_2_ can be dissociated over metal surfaces to generate active hydrogen, and a subsequent hydrogenation occurs with reactant molecules [53]. From this perspective, a catalyst with smaller metal nanoparticles should possess more accessible catalytically active sites and, consequently, exhibit higher hydrogenation activity. In this study, the Ni/ZrO_2_ (P) catalyst exhibited stronger metal-support interaction than the Ni/ZrO_2_ (H) catalyst because of its greater number of oxygen vacancies and low-coordinated oxygen ions on the surface. Thus, smaller Ni particles were obtained on Ni/ZrO_2_ (P) catalyst. Furthermore, the H_2_-TPD result illustrates that the Ni/ZrO_2_ (P) catalyst possessed a higher metal Ni surface area and more hydrogen activation sites. In the MA hydrogenation reaction, the Ni/ZrO_2_ (P) catalyst also exhibited a higher C=C hydrogenation activity, which was predictable and understandable. However, the C=O hydrogenation activity of the Ni/ZrO_2_ (P) catalyst was much lower than that of the Ni/ZrO_2_ (H) catalyst. A 43.5% yield of GBL was obtained over the Ni/ZrO_2_ (H) catalyst, while 2.8% yield only (of GBL) was obtained over the Ni/ZrO_2_ (P) catalyst under the same reaction conditions. Similar behavior was observed when we investigated the catalyst performances of MA hydrogenation over the Ni/ZrO_2_(P) and Ni/ZrO_2_(H) catalysts with 5 wt % nickel loading. The Ni crystalline size for Ni/ZrO_2_ (P) catalyst with 5 wt % nickel loading was 9 nm while that for Ni/ZrO_2_ (H) catalyst was 10 nm (Figure S1). Two catalyst possessed the similar crystalline size, whereas their catalytic performance was quite different (Table S1). The yield of GBL for Ni/ZrO_2_ (P) catalyst with 5 wt % nickel loading was still very low, only 2.1%. While that for Ni/ZrO_2_ (H) catalyst with 5 wt % nickel loading was 20.8%. The above results demonstrate that the C=O hydrogenation activities of the catalysts did not correlate well with the hydrogen activating ability of the catalyst or the Ni crystalline size. This strongly suggests that, in addition to the catalytic ability of metal Ni, other factors influenced the C=O hydrogenation activity for the studied ZrO_2_-supported nickel catalyst system.

The results of in-situ FT-IR of adsorbed cyclohexanone showed that the Ni/ZrO_2_ (H) catalyst was able to adsorb and activate C=O groups effectively, whereas the Ni/ZrO_2_ (P) catalyst exhibited extremely weak adsorption and activation abilities for the same groups. The results of this study’s experiments suggest that the superior catalytic activity of the Ni/ZrO_2_ (H) catalyst in C=O hydrogenation can be attributed to its effective activation of the C=O group in the SA molecule. Hu et al. found that a large number of oxygen vacancies on Mn-containing spinel-supported copper catalyst contributed to the C=O hydrogenation [54]. Han et al. and Manyar et al. also observed similar results [55,56]. In our study, the Raman and XPS characterization results showed that the electronic properties of oxygen vacancies were significantly different on the surface of the Ni/ZrO_2_ (P) and the Ni/ZrO_2_ (H) catalyst. Surface oxygen vacancies on the Ni/ZrO_2_ (P) catalyst exhibited relatively electron-rich properties while those on the Ni/ZrO_2_ (H) catalyst showed relatively electron-deficient properties. Compared to relatively electron-rich oxygen vacancies, relatively electron-deficient oxygen vacancies were more likely to interact with lone pair electrons on the oxygen atoms of C=O groups, and so C=O groups could be activated. Given this, it can be deduced that relatively electron-deficient surface oxygen vacancies play a key role in promoting the hydrogenation of C=O groups. As concerns the Ni/ZrO_2_ (H) catalyst, the oxygen vacancies promoted by Ni^0^ located at the Ni/ZrO_2_ interface exhibited lower charge densities and were more likely to adsorb and activate C=O groups in SA, thereby weakening the C=O bonds and lowering the energy requirement for hydrogenation and promoting C=O hydrogenation through their synergy with neighboring Ni particles.

**Figure 11:**
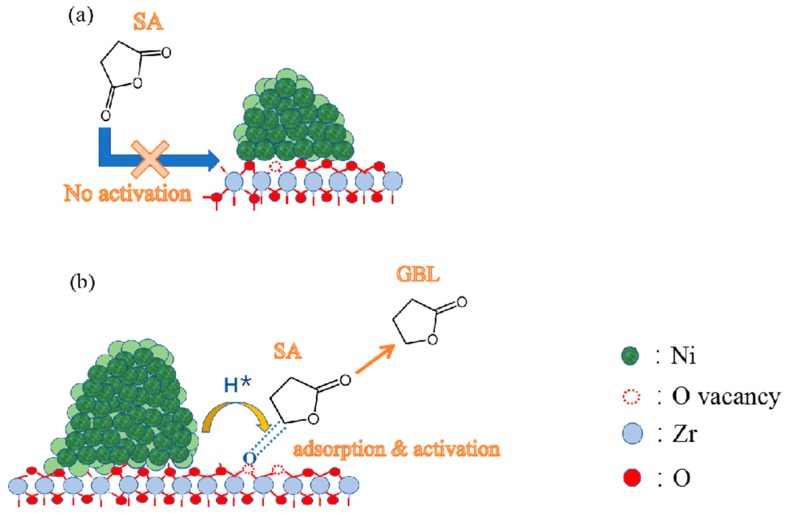
(**a**) Structure of nickel particles on the ZrO_2_ (P). (**b**) Structure of nickel particles on the ZrO_2_ (H) and hydrogenation of SA on the Ni/ZrO_2_ (H).

Based on the above results, a plausible, simplified mechanism for C=O hydrogenation over Ni/ZrO_2_ is proposed (Figure 11). Relatively electron-deficient oxygen vacancies on surface of Ni/ZrO_2_ (H) catalyst activate the C=O bonds by accepting a lone pair of electrons from the oxygen atom of the C=O bonds, and thereby weakening the C=O bonds. Ni particles distributed near the relatively electron-deficient oxygen vacancies on the Ni/ZrO_2_ (H) catalyst dissociated H_2_ to produce active hydrogen and finish the C=O hydrogenation with the synergism of oxygen vacancies. As long as the relatively electron-rich oxygen vacancies on surface of Ni/ZrO_2_ (P) catalyst cannot effectively activate the C=O bond, it will be difficult to achieve the C=O hydrogenation.

## 5. Conclusions

This work showed an effective strategy for manipulating product selectivity in MA hydrogenation through regulating surface structures of Ni/ZrO_2_ catalysts. The ZrO_2_ (P) support, with more oxygen vacancies and low-coordinated oxygen ions on its surface, exhibited much stronger interactions with nickel species, which resulted in a small number of nickel species entering into oxygen vacancies and, thus, forming a Ni-O-Zr structure. This led to a decrease in the oxygen vacancy concentration and an increase of the average charge densities of the oxygen vacancies, which then produced high selectivity towards SA from MA hydrogenation. However, ZrO_2_ (H), which had stable oxygen ions and fewer oxygen vacancies, showed weaker interactions with nickel species, resulting in large Ni particles being poorly dispersed on the Ni/ZrO_2_ (H) and relatively electron-deficient oxygen vacancy generation promoted by Ni^0^ particles. Thus, the Ni/ZrO_2_ (H) exhibited a high selectivity towards GBL. The high C=O hydrogenation activities for the Ni/ZrO_2_ (H) catalyst were attributed to the surface synergy between active metallic nickel species and relatively electron-deficient oxygen vacancies. These conclusions offer a new strategy for the design of high-efficiency selective hydrogenation catalysts applied to α, β-unsaturated aldehyde and ketone hydrogenation reactions by modulating the surface structure of ZrO_2_ supports.

## Figures and Tables

**Table 1:** A summary of metal content, specific surface area and pore diameter, calculated tetragonal phase content, and crystalline size of the ZrO_2_ (P) and ZrO_2_ (H) supports and their corresponding supported nickel catalysts.

Catalysts	Metal Content ^[a]^ [wt %]	S_BET_ [m^2^g^−1^]	Pore Diameter [nm]	Tetragonal Phase ^[b]^ [%]	Tetragonal Phase ^[c]^ [%]	Tetragonal Phase ^[d]^ [%]	Crystalline Size of ZrO_2_^[e]^ [nm]	Crystalline Size of NiO ^[f]^ [nm]	Crystalline Size of Ni ^[g]^ [nm]
ZrO_2_ (P)	-	98	7.1	48	distorted	23	16	-	-
NiO/ZrO_2_ (P)	-	73	5.8	47	-	disordered	17	16	-
Ni/ZrO_2_ (P)	9.42	53	3.5	46	-	-	17	-	18
ZrO_2_ (H)	-	71	3.8	39	35	11	10	-	-
NiO/ZrO_2_ (H)	-	58	3.7	41	-	distorted	10	26	-
Ni/ZrO_2_ (H)	9.39	25	3.6	41	-	-	10	-	40

**^[a]^** Measured by inductively coupled plasma (ICP). **^[^^b]^** Calculated using the Rietveld method from the X-ray powder diffraction data. Calculated using the Raman data at a **^[^^c]^** 325 nm and **^[^^d]^** 532 nm excitation wavelengths. Calculated using the Scherrer equation from the^**[**^**^e]^** (-111) plane of m-ZrO_2_, **^[^^f]^** (012) plane of NiO, and **^[^^g]^** the (111) plane of Ni.

**Table 2:** The binding energy of O 1s lines and the corresponding surface atomic concentration of ZrO_2_ supports and Ni/ZrO_2_ catalysts.

Samples	Binding Energy (eV)				*I* (%)				
	BE_1_	BE_2_	BE_3_	BE_4_	*I*_1_ (%)	*I*_2_ (%)	*I*_3_ (%)	*I*_4_ (%)	*I*_2_ + *I*_3_ (%)
ZrO_2_ (P)	529.7	531.0	531.9	532.8	62.9	21.2	11.9	4.0	33.1
ZrO_2_ (H)	529.8	530.8	531.7	532.8	71.0	11.6	12.9	4.5	24.5
Ni/ZrO_2_ (P)	529.8	530.8	531.7	532.8	71.8	10.1	14.6	3.5	24.7
Ni/ZrO_2_ (H)	529.8	531.0	531.9	532.8	70.5	13.4	13.0	3.1	26.4

**Table 3:** Binding energy of Ni 2p lines and the corresponding surface concentration of Ni/ZrO_2_ catalysts.

Samples	Ni^0^ (Ni 2P_3/2_)	NiO (Ni 2P_3/2_)	Ni^2+^ (Ni 2P_3/2_)	C(Ni^0^) (%)	C(NiO) (%)	C(Ni^2+^) (%)
Ni/ZrO_2_ (P)	852.2	853.9	855.6	57.8	22.5	19.7
Ni/ZrO_2_ (H)	852.2	853.0	855.1	58.4	19.2	22.4

**Table 4:** MA conversion and GBL selectivity over the Ni/ZrO_2_ (P) and Ni/ZrO_2_ (H) catalysts under different temperature and pressure conditions for 480 min.

Catalysts	Temperature (°C)	Pressure (MPa)	Conv. (%)	GBL Selec. (%)
Ni/ZrO_2_ (P)	210	5	100	2.8
	240	5	100	4.9
	210	7	100	3.2
Ni/ZrO_2_ (H)	210	5	100	43.5
	240	5	100	60.6
	210	7	100	46.2

## Supplementary Materials

The Supplementary Materials are available online at https://www.mdpi.com/2079-4991/9/3/406/s1. Figure S1: XRD patterns of Ni/ZrO_2_ (P) and Ni/ZrO_2_ (H) catalysts with 5 wt % nickel loading, Table S1: The catalytic performance in MA hydrogenation of Ni/ZrO_2_ catalysts with 5 wt % nickel loading.
