# Supplementary material for: Synergistic Effect of Oxygen Vacancies and Ni Species on Tuning Selectivity of Ni/ZrO2 Catalyst for Hydrogenation of Maleic Anhydride into Succinic Anhydride and γ-Butyrolacetone
Source: Nanomaterials (Basel). 2019 Mar 11;9(3):406. doi: 10.3390/nano9030406 (PMC6474034; doi:10.3390/nano9030406)
Supplement: Supplementary file 1 [file nanomaterials-09-00406-s001.pdf]

Synergistic Effect of Oxygen Vacancies and Ni Species on Tuning Selectivity of Ni/ZrO_2 Catalyst for Hydrogenation of Maleic Anhydride into Succinic Anhydride and γ-Butyrolacetone

Lili Zhao [1], Jianghong Zhao [1], Tianjie Wu [1], Min Zhao [2], Wenjun Yan [2], Yin Zhang [1], Haitao Li [1], Yongzhao Wang [1], Tiancun Xiao [3,*] and Yongxiang Zhao [1,*]

1. Engineering Research Center of Ministry of Education for Fine Chemicals, School of Chemistry and Chemical Engineering, Shanxi University, Taiyuan 030006, China; lzhao@sxu.edu.cn (L.Z.); zhaojianghong@sxu.edu.cn (J.Z.); 201722907009@email.sxu.edu.cn (T.W.); sxuzhy@sxu.edu.cn (Y.Z.); htli@sxu.edu.cn (H.L.); catalyst@sxu.edu.cn (Y.W.)
2. Institute of Coal Chemistry, Chinese Academy of Sciences, Taiyuan 030001, China; zhaomin@sxicc.ac.cn (M.Z.); yanwenjun@sxicc.ac.cn (W.Y.)
3. Inorganic Chemistry Laboratory, Oxford University, Oxford, OX1 3QR, UK

* Correspondence: xiao.tiancun@chem.ox.ac.uk (T.X.); yxzhao@sxu.edu.cn (Y.Z.)

Supporting Information

S1. XRD patterns of Ni/ZrO_2 (P) and Ni/ZrO_2 (H) catalysts with 5wt% nickel loading

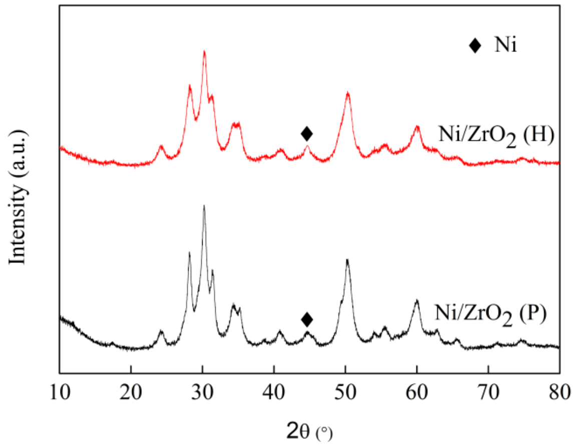

Figure S1. XRD patterns of Ni/ZrO_2 (P) and Ni/ZrO_2 (H) catalysts with 5wt% nickel loading

S2. The catalytic performance in MA hydrogenation of Ni/ZrO_2 catalysts with 5wt% nickel loading

Table S1. The catalytic performance in MA hydrogenation of Ni/ZrO_2 catalysts with 5wt% nickel loading

catalysts	Crystalline size of Ni (nm)	Conversion of MA (%)	Selectivity of GBL (%)
Ni/ZrO_2 (P)	9	100	2.1
Ni/ZrO_2 (H)	10	100	20.8
